# Effect of Root Storage and Forcing on the Carbohydrate and Secondary Metabolite Composition of Belgian Endive (*Cichorium intybus* L. Var. *foliosum*)

**DOI:** 10.1021/acsfoodscitech.2c00182

**Published:** 2022-09-23

**Authors:** Jeroen van Arkel, Anna Twarogowska, Yannah Cornelis, Tania De Marez, Jasper Engel, Peter Maenhout, Ric C. H. de Vos, Jules Beekwilder, Bart Van Droogenbroeck, Katarina Cankar

**Affiliations:** † Wageningen University and Research, BU Bioscience, Wageningen Plant Research, Droevendaalsesteeg 1, 6708PB Wageningen, The Netherlands; ‡ ILVO, Flanders Research Institute for Agriculture, Fisheries, and Food, Technology and Food Science Unit, Brusselsesteenweg 370, BE-9090 Melle, Belgium; § Praktijkpunt Landbouw Vlaams-Brabant vzw, Blauwe Stap 25, BE-3020 Herent, Belgium; ∥ Inagro vzw, Ieperseweg 87, BE-8800 Rumbeke-Beitem, Belgium

**Keywords:** Belgian endive, Cichorium intybus L. var. foliosum, witloof, storage, forcing, inulin, chlorogenic acid, chicoric acid, lactucin, lactucopicrin

## Abstract

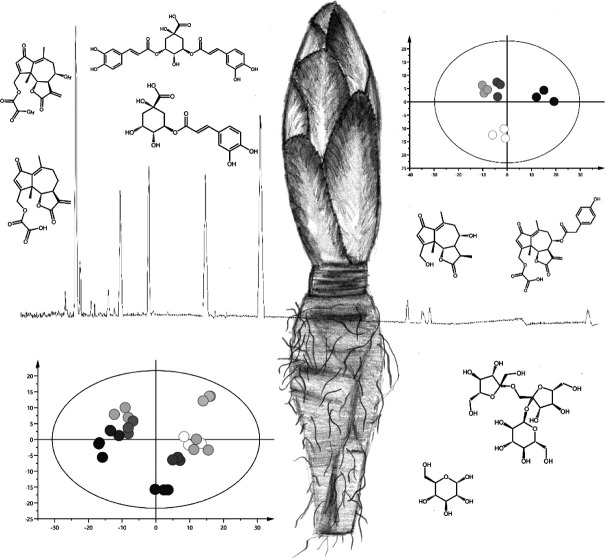

Belgian endive is grown in a two-step cultivation process that involves growing of the plants in the field, cold storage of the taproots, and a second growth period in dark conditions called forcing to yield the witloof heads. In this study, the changes in the carbohydrate content and the secondary metabolite composition were studied in different tissues of Belgian endive during the cultivation process. Belgian endive heads contain between 336–388 mg/g DW of total soluble carbohydrates, predominantly fructose and glucose. The heads also contain phenolic compounds and terpenoids that give Belgian endive its characteristic bitter taste. The terpenoid and phenolic compound composition of the heads was found to be constant during the cultivation season, regardless of the root storage time. In roots, the main storage carbohydrate, inulin, was degraded during storage and forcing processes; however, more than 70% of total soluble carbohydrates remained unused after forcing. Additionally, high amounts of phenolics and terpenoids were found in the Belgian endive taproots, predominantly chlorogenic acid, isochlorogenic acid A, and sesquiterpene lactones. As shown in this study, Belgian endive taproots, which are currently discarded after forcing, are rich in carbohydrates, terpenes, and phenolic compounds and therefore have the potential for further valorization. This systematic study contributes to the understanding of the carbohydrate and secondary metabolite metabolism during the cultivation process of Belgian endive.

## Introduction

*Cichorium intybus* is a perennial plant, which is grown as a vegetable (*C. intybus* L. var. *foliosum*) and is appreciated for its bitter taste. Examples are Belgian endive and radicchio. Another variety of chicory *C. intybus* L. var. *sativum*, also called industrial chicory, is cultivated for the extraction of the food fiber inulin. Belgian endive or witloof is an important vegetable crop in Belgium, France, and the Netherlands. The estimated total yearly EU production of Belgian endive amounts to 350,000 ton.^1^

**Figure 1:**
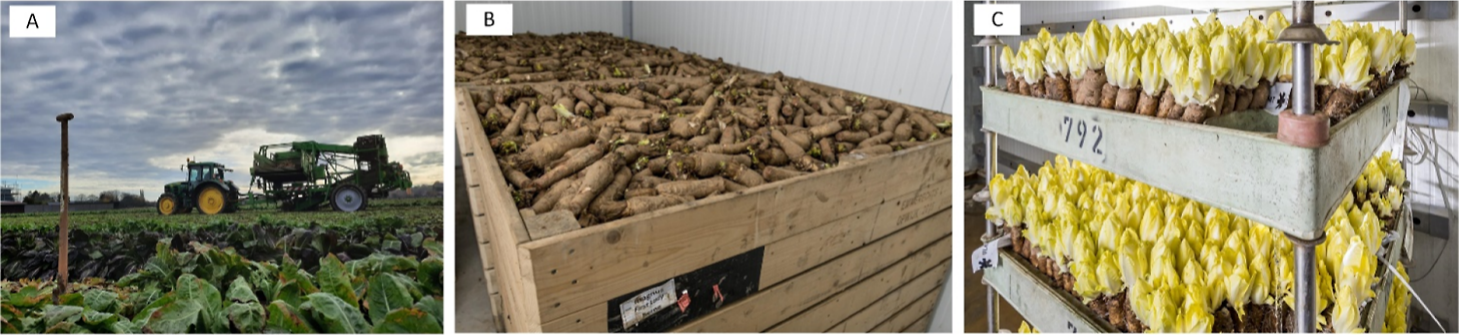
**Cultivation process of Belgian endive.** (A) Harvesting of taproots after cultivation of Belgian endive in the field (photograph by Yannah Cornelis), (B) Cold storage of Belgian endive taproots (photograph by Lander Loeckx), (C) Second growth period or forcing involves hydroponic cultivation of taproots in darkness for production of Belgian endive heads (photograph by Lander Loeckx).

For the production of Belgian endive two cultivation phases are needed (Figure 1). First, the plants are grown on an open field for the production of the taproot. In autumn, the taproot is harvested and stored in cold conditions between −1 and 4 °C at a high relative humidity of 95–98%. The cold storage can last up to 12 months, depending on the cultivar, and is needed for the outgrowth of the head.^2^ The second cultivation process called forcing starts with defrosting of the taproots, that are subsequently cultivated on hydroponics in specialized chambers in the dark at elevated temperatures. The temperature choice depends on the Belgian endive variety used. After 3 weeks, a blanched, densely packed head is formed on the taproot. The head is harvested, and the remaining forced taproot is discarded as waste. The storage conditions of the taproots can influence the quality of the produced head.^3,4^ However, after many years of optimization, the current hydroponics-based production is robust and guarantees a year-round production of fresh Belgian endive.

The discarded forced taproots form the major waste stream of the Belgian endive cultivation, constituting more than half of the harvested biomass. In Europe approximately 300,000–400,000 tons of forced roots (FR) are produced annually.^1^ Currently, the roots do not have a high value and are discarded or used as animal feed. The Belgian endive taproot is rich in carbohydrates. Inulin, a linear polymer of fructose linked to a terminal glucose moiety, is the major storage carbohydrate in chicory roots.^5^ In industrial chicory, about 70% of the taproot dry weight (DW) is composed of inulin.^6,7^ The degree of polymerization of inulin typically ranges from 2 to 60; however, fractions with a low degree of polymerization are removed when inulin is extracted to be used as food fibre.^8^

In Belgian endive, typically inulin of low degree of polymerization is present; therefore, its taproots are not exploited as a source of dietary fibre.^9^ During storage and forcing of the Belgian endive taproots, the quantity of inulin is decreased as it is converted into monosaccharides to be used for the basic metabolism in the storage phase, as well as for the development of the head in the forcing phase.^10^

**Figure 2:**
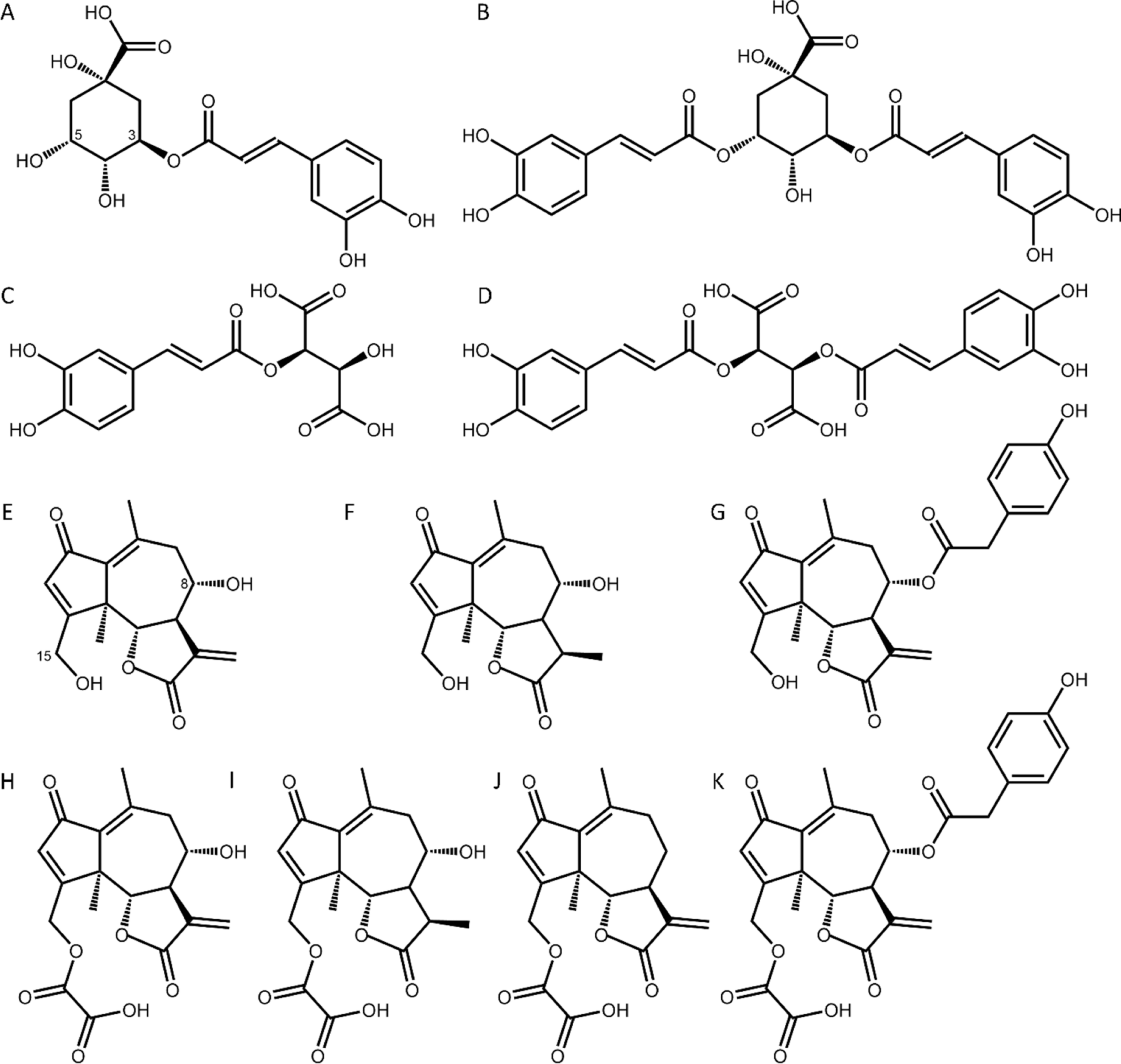
**Sesquiterpene lactones and phenolic compounds of Belgian endive**. (A) chlorogenic acid, (B) isochlorogenic acid A, (C) caftaric acid, (D) chicoric acid, (E) lactucin, (F) dihydrolactucin, (G) lactucopicrin, (H) lactucin 15-oxalate, (I) dihydrolactucin 15-oxalate, (J) 8-deoxylactucin 15-oxalate, and (K) lactucopicrin 15-oxalate.

In addition to carbohydrates, the taproots of both industrial chicory and Belgian endive are rich in phenolic compounds and terpenoids (Figure 2).^11,12^ Industrial chicory is particularly rich in hydroxycinnamic acid derivatives, including chlorogenic acid (3-caffeoylquinic acid, 3-CQA) and isochlorogenic acid A (3,5-dicaffeoylquinic acid, di-CQA). In addition, two caffeic acid esters of tartaric acid, that is, caftaric acid and chicoric acid, are accumulating in industrial chicory but are predominantly found in the leaves.^13^ In a study on different varieties of *C. intybus,* it was shown that also Belgian endive contains phenolic acids, namely chlorogenic acid, caftaric acid, and chicoric acid, like many other leafy chicory vegetables.^14^ The phenolic compounds are thought to be involved in the plant’s defense against biotic and abiotic stress.^15^ Their proposed biological functions in plants include antibacterial, antiviral, and antifungal activities, and they also serve as antioxidants and oxygen free radical scavengers. In particular, the di-CQA’s have potent antioxidant capacity, for example, in coffee (*Coffea canephora*)^16^ and are increased upon UV exposure, as shown in* Vitis vinifera* leaves^17^ and artichoke.^18^ Also, for humans, the phenolics have potential health effects. Epidemiological studies suggest correlations between the intake of high levels of phenolics and the prevention of some diseases,^19^ such as thrombose and inflammation.^20^ It is generally believed that the health-promoting activities of the phenolics are due to the ability to scavenge free radicals, as shown by Kono et al. for chlorogenic acid and caffeic acid.^21^

The roots and leaves of *C. intybus* plants are also rich in sesquiterpene lactones (STLs).^22^ STLs are sesquiterpenes consisting of a 15-carbon skeleton with a characteristic lactone ring containing a conjugated exomethylene group (α-methylene-γ-lactone). The major STLs reported in chicory are the guaianolide-type STLs lactucin, 8-deoxylactucin, and lactucopicrin, predominantly present in their oxalated form.^22^ In plants, the STLs function in resistance against several pathogens^23−25^ and play a role in resistance to herbivore attack.^26^ The oxalate esters of STLs are thought to have an antifeedant role. As they are unstable and may decompose upon tissue damage, both oxalic acid and the STLs will be released and may contribute to the antifeedant properties.^22^ In chicory, as well as in many other plant species, STLs are stored in latex in specialized organs present in the leaves and the roots called laticifers.^27^ STLs are known to have several health benefits for humans.^28^ The sesquiterpene α-methylene-γ-lactone-ring of the molecules is thought to be essential for their functionality. This ring structure facilitates the alkylation of cellular thiol-groups, found in, for example, glutathione and in cysteine-containing proteins. In this manner, STLs influence the functioning of thiols in regulating cellular mechanisms, for example, by sensitizing cancer cells for chemical treatments^29,30^ or lowering blood pressure in humans.^31^ Chicory STLs show analgesic and sedative activities in mice.^32^ An intervention study showed the anti-inflammatory potential of chicory STLs against osteoarthritis in humans.^33^

In this work, metabolic changes in carbohydrate, phenolic compound, and sesquiterpene lactone profiles were studied in Belgian endive taproots and heads during its cultivation cycle, including field harvest, storage, and forcing. The carbohydrate and secondary metabolite composition of heads, which are used as a vegetable, was studied through the cultivation season of 1 year. Additionally, the metabolic composition of taproots, which are currently discarded after forcing, was studied to determine the potential for their upcycling.

## Materials and Methods

### Plant Material

Belgian endive (*C. intybus* L. var. *foliosum*) cultivars “Sweet Lady” and “Flexine” were grown on experimental fields at Praktijkpunt Landbouw Vlaams-Brabant vzw (Herent, Belgium) in year 2019. Both cultivars are considered late cultivars that can produce high-quality heads after prolonged storage for up to 1 year. Field cultivation was performed according to general crop husbandry comparable to commercial fields. The USDA soil classification for this location was Alfisol.^34^ In October 2019, the taproots were harvested, cleaned, and stored in wooden containers in storage compartments at −1 °C. Samples of taproots (field roots) and field-grown leaves (field leaves) were sampled at harvest, processed as described below, and stored until metabolite analysis. Second cultivation, also called forcing, was performed after 1, 3, 6, and 12 months of root storage for cultivar “Sweet Lady” and after 12 months for cultivar “Flexine.” In parallel, the Belgian endive cultivar “Sweet Lady” was cultivated, stored for 6 months, and forced at a second location at Inagro in Rumbeke-Beitem, Belgium. Taproots were defrosted, placed in trays, and cultivated in hydroponic cultures in darkness for 3 weeks at 15–16 °C. The hydroponics solution was supplemented with 50% Kristalon Label Blue (Yara), 25% potassium nitrate, and 25% calcium nitrate to a final EC (electrical conductivity) of about 1.8 mS/cm at pH = 6.8. Non forced roots (NFR) are roots that were defrosted and sampled before the second cultivation phase started. Forced roots (FR) are roots collected after the cultivation in hydroponic culture and harvesting of Belgian endive heads. FR, NFR, and heads were sampled for carbohydrate- and secondary metabolite analysis in triplicate. For each replicate, 20 roots or heads were collected, washed under cold tap water, and cut into 1 cm^3^ cubes using a Robot Coupe cutter, model CL 50 Ultra (Robot Coupe, Mont-Sainte-Geneviève, Belgium). The samples were subsequently freeze-dried using an Epsilon 2–10 D LSC freeze dryer (Martin Christ, Osterode am Harz, Germany) and milled to a fine powder using an Ultra centrifugal mill ZM 200 (Retsch, Haan, Germany). The resulting dry powders were stored in airtight sealed aluminum-foil-laminated plastic bags (Rapak Corporation, Rugby, United Kingdom) at room temperature until analysis.

### Carbohydrate Analysis

Carbohydrate analysis was performed to determine the concentrations of free fructose, sucrose, and glucose, total carbohydrate content, inulin content, and inulin mean degree of polymerization (mDP) in all samples of the cultivar “Sweet Lady” cultivated at the location Herent. The analysis was performed according to van Waes et al.^35^*.* In short, free sugars were extracted from the freeze-dried plant material in water at 85 °C for 1 h, the extract was filtered, and the free glucose, fructose, and sucrose concentrations were determined by ion chromatography using a Dionex ICS 3000 with pulsed amperometric detection on a 250 × 4 mm Dionex CarboPac PA1 column with 100 mM sodium hydroxide as eluent with an isocratic flow of 1 mL/min. Quantification of the three sugars was performed by comparison of peak areas to a standard curve prepared from authentic standards.

The total carbohydrate and inulin content was analyzed in hydrolyzed extracts of the freeze-dried plant material. Hydrolysis was performed in water with the addition of 5 mL of 3 M hydrochloric acid at 85 °C for 1 h. After cooling, neutralization, and filtration, the samples were analyzed with a Dionex ICS 3000 chromatography system on a Metacarb 67C (Agilent Technologies) column at 90 °C with deionized water as a mobile phase at a flow rate of 0.5 mL/min. Peak detection was performed using a Perkin Elmer 2414 refractive index detector. The total carbohydrate content was calculated as the sum of fructose and glucose in the hydrolyzed plant extract divided by 1.1. The inulin content was calculated as the total carbohydrate content from which the concentrations of free glucose, fructose, and sucrose were subtracted. The mDP was calculated as the ratio of fructose to glucose in the hydrolyzed plant extract.^35^

### Metabolite Analysis and Profiling

Semipolar metabolites were analyzed by LC–MS.^36^ In brief, 100 mg (+/–3 mg) of freeze-dried plant material was extracted using 1400 μL of 80% methanol supplemented with 0.13% formic acid. The samples were incubated for 15 min in an ultrasonic bath. Next, the debris was separated from the extract by centrifugation. LC–MS analysis was performed using an LC-PDA-LTQ-Orbitrap FTMS system (Thermo Scientific), which consisted of an Acquity HPLC with an Acquity eLambda photodiode array detector (220–600 nm) connected to an LTQ/Orbitrap XL hybrid mass spectrometer equipped with an electrospray ionization source (ESI). Chromatographic separation was on a reversed-phase column (Luna C18/2,3 μm 2.0 × 150 mm; Phenomenex, USA) at 40 °C. Degassed eluent A [ultra-pure water: formic acid (1000:1, v/v)] and eluent B [acetonitrile: formic acid (1000:1, v/v)] were used at a flow rate of 0.19 mL min^–1^. A linear gradient from 5 to 75% acetonitrile (v/v) in 45 min was applied, which was followed by 15 min of washing and equilibration. FTMS full scans (*m*/*z* 90.00–1350.00) were recorded with a mass resolution of 60,000 FWHM.

Quantification of selected metabolites was performed by comparison of LCMS peak areas to a standard curve prepared from authentic standards of chlorogenic acid (Sigma-Aldrich), caftaric acid (Sigma-Aldrich), chicoric acid (Sigma-Aldrich), isochlorogenic acid A (Sigma-Aldrich), lactucin (Extrasynthese), and lactucopicrin (Extrasynthese). For compounds for which authentic standards are not available, the MS detector response (ion counts/scan) of the base peak in the negative ionization mode is presented as the relative peak area.

### LC–MS Data Analysis

For the targeted metabolite analysis, mass-specific peak areas were integrated using Xcalibur software version 4.1 (Thermo Scientific). For untargeted data processing, the LC–MS data files were processed using Metalign software^37^ (http://www.metalign.nl). Baseline correction and noise determination were performed, and successively, the *m*/*z* values were aligned. After removing low and inconsistent signals, that is, present in <3 samples or with an ion intensity lower than 5000 in all samples, the remaining mass signals were subjected to MSClust software^38^ in order to cluster mass signals derived from the same metabolite based on their corresponding retention time and abundance pattern across samples. This resulted in the relative peak intensities of 554 and 295 mass clusters, respectively, for the data generated on root and head samples of the cultivar “Sweet Lady,” respectively, each representing a putative metabolite present in at least 3 samples.

### Statistical Methods

Principal component analysis (PCA) was performed on the log2-transformed and mean-centered data matrix using the software package SIMCAp (version 17.02, Umetrics). Statistical analysis was performed on individual metabolites and attributes that were further studied using SPSS software (version 25 for Windows, IBM) and R (version 4.0.3). Two-way analysis of variance (ANOVA) was carried out to assess the differences in metabolite levels between the tissues (NFR, FR, and head), the effect of forcing on the roots, and the effect of root storage time on the roots and head in “Sweet Lady.” The model comprised fixed effects for tissue type, storage time (1, 3, 6, and 12 months of root storage), and their interaction. An F-test employing type III sums of squares was used to assess the significance of each effect. In case of a significant interaction effect (*p* ≤ 0.05), we followed up with Bonferroni posthoc tests between time points within each tissue type and between tissues within each timepoint. For those compounds with a nonsignificant interaction (*p* > 0.05), the term was dropped from the model, and posthoc tests (Tukey’s test) focused on differences between levels of one factor only (equally averaged over the levels of the other factor). Proportions, such as the total carbohydrate, were analyzed analogously using logistic regression with a beta distribution using R package GlmmTMB. A type III Wald test was used to assess the significance of main effects and interactions, such as tissue type and storage time, for all components using R package Car. For the mDP, a two-way ANOVA was performed to reveal the interaction between tissue type and storage time for these compounds. Finally, the R package Emmeans was used to carry out pairwise comparisons by an approximate *t*-test in combination with the multiple testing corrections mentioned above (Bonferroni, Tukey, or Dunnett).

To study the influence of cold storage on the roots more specifically, field root samples of the cultivar “Sweet Lady” were compared to the stored roots at *t* = 1, 3, 6, and 12 months using a two-sided Dunnet’s test.

Next, the differences in metabolite levels between the two cultivars ‘Sweet Lady and “Flexine” were assessed by means of a two-way ANOVA with fixed effects for the cultivar, tissue type, and their interaction. Note that this analysis focused on samples taken at 12 months of root storage only. Testing was carried out analogous to what was described above. Finally, two-way ANOVA with posthoc testing was used to carry out a comparison between the first and second growing location of “Sweet Lady” after 6 months of root storage. The model comprised fixed effects for location, tissue type, and their interaction.

## Results and Discussion

### Process of Storage and Forcing Mobilizes the Storage Carbohydrates in Belgian Endive Roots

The taproot of *C. intybus* species serves as the storage organ where carbohydrates are stored to overcome the winter and to ensure a quick regrowth of the leaves and flowering in spring or for etiolated head formation in the cultivation of Belgian endive. The main storage carbohydrate in taproots is inulin. In this study, the soluble carbohydrate content was studied in different tissues of Belgian endive: field leaves, field roots, NFR collected before the second cultivation phase in hydroponic culture, and the produced Belgian endive heads and FR collected after harvesting Belgian endive heads. The forcing process was initiated after the taproots had been stored for 1, 3, 6, or 12 months, and the effect of storage duration on soluble carbohydrates was studied.

**Figure 3:**
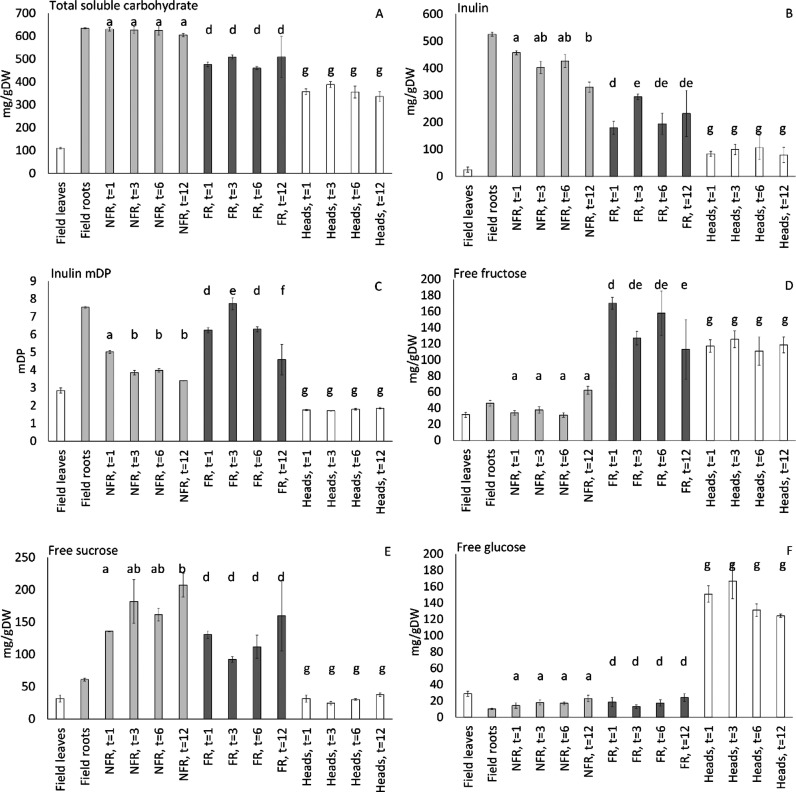
**Carbohydrate content of Belgian endive tissues during storage and forcing.** Carbohydrate composition is shown for field leaves (white), NFR (light grey), FR (dark grey), and heads (white) at different times of root storage (*t* = 1, 3, 6, and 12 months). The panels represent: (A) total soluble carbohydrate (mg/g DW), (B) inulin content (mg/g DW), (C) inulin mDP, (D) free fructose (mg/g DW), (E) free sucrose (mg/g DW), and (F) free glucose (mg/g DW). The error bars represent the standard errors. The letters in the figure indicate the significance of the Bonferroni posthoc test, focusing on differences between samples of different time points of the same tissue type.

The total soluble carbohydrate content in the field roots is 634 mg/g DW (Figure 3). The total carbohydrate content in nonforced taproots was stable during root storage at −1 °C for up to 12 months. Upon forcing, the total carbohydrate level decreased to between 476 and 509 mg/g DW, showing that more than 70% of the initial total carbohydrates are still left in the roots after Belgian endive heads have been harvested. The level of statistical significance of the effects of storage time, tissue, and their interaction is given in Supporting Table S1. The statistical comparison between field roots and stored roots is presented in Supporting Table S2.

The major storage carbohydrate in the taproots is inulin, which constitutes 525 mg/g DW directly after field harvest. Upon storage, the inulin content in NFR gradually decreases to 330 mg/g DW after 12 months, accompanied by an increase in sucrose levels to 207 mg/g DW. Most probably, the inulin is degraded due to fructan exohydrolase (FEH) enzyme depolymerization activity that is induced at harvest.^6,39^ This enzyme releases fructose from the inulin pool, but surprisingly, no increase in fructose was observed. Sucrose content, however, was four-fold increased upon storage. Most probably, the released fructose was converted into sucrose by the activity of fructokinase forming fructose 6-phosphate and successive enzymes initiating sucrose cycling.^40^ The released carbohydrates are not metabolized as the total carbohydrate levels do not change. The sugars released from inulin are presumably acting as antifreeze during cold storage.^41,42^

An additional decrease in inulin content in taproots is observed upon forcing when the carbohydrates are mobilized to form the head. Simultaneously, the level of free fructose in FR is increased three-fold as compared to NFR. This finding is supported by a previous study where the effect of forcing was studied on the different inulin polymers^39^ and the level of total carbohydrates in the roots.^43^ The inulin mDP measured in roots was low, ranging from 3 to 8. This is in line with previous observation of inulin mDP in Belgian endive.^44^ Owing to low mDP, Belgian endive roots are not suitable for inulin extraction as a dietary fiber.^9^ While inulin content decreases during forcing, unexpectedly, the mDP value seems to be increased upon forcing. This is most probably the result of the formation of inulo-*n*-oses, polymers of fructose not containing the terminal glucose, which disturb the mDP calculation, as previously discussed by van Arkel.^6^

The Belgian endive heads contain between 336 and 388 mg/gDW of total soluble carbohydrates (Figure 3). In contrast to the taproot, low amounts of inulin are found in the head. In comparison to the root, relatively lower amounts of sucrose and higher amounts of glucose and fructose were found in the heads. The low levels of sucrose and equal amounts of fructose and glucose in the head can most probably be explained by the activity of invertase found in leafy tissue of plants.^45^ Indeed, expression of vacuolar invertase has been reported in the Belgian endive head as well.^46^ The duration of storage of taproots did not influence the carbohydrate profiles found in the head. This supports the rather constant head quality throughout the cultivation season, as the free sugar levels might influence the palatability of the head. The heads show a high carbohydrate content compared to the field leaves. This difference could be explained by the functional differences between the two tissues, the field leaves are source organs for sugars that are designed to export the sugars to the roots. The leaves of the Belgian endive head are a sink organ that uses the carbon from the taproot for its fast outgrowth.

Belgian endive heads are consumed as a vegetable, while the roots are discarded after forcing. As shown here, the taproots contain 634 mg/g DW of soluble carbohydrates at harvest, and after forcing, the carbohydrate content is only partially reduced to about 476–509 mg/g DW. In a previous study, the amount of stored sugar was not deemed limiting for the quality and yield of the crop.^47^ However, our finding is in contrast with an earlier study performed on the storage of chicory taproots for 6 weeks, where a nearly complete breakdown of inulin was found upon forcing.^48^ An explanation for the observed difference could be the storage temperature: in our experiments, a temperature of −1 °C was applied in contrast to the +3 and +5 °C that Rutherford and Weston used. We demonstrate here that after storage of roots at −1 °C, high amounts of soluble carbohydrates are still present in the FR. The free sugars and inulin left in the FR, therefore, could be an interesting source for the production of biogas or platform chemicals, such as HMF.^49^

### Changes in the Secondary Metabolite Profile of Belgian Endive upon Storage and Forcing

**Figure 4:**
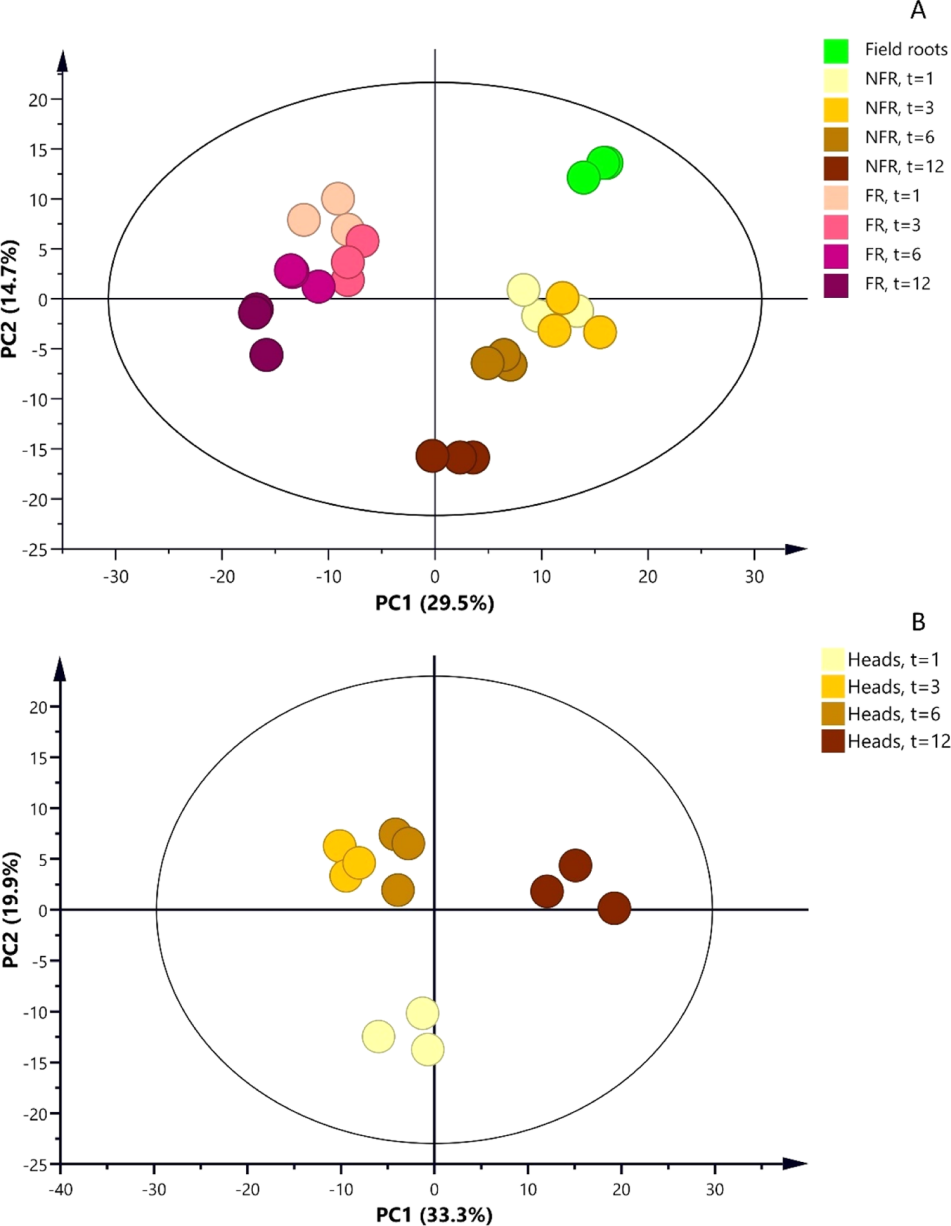
**PCA score plot of the LC–MS profiles of Belgian endive root and head samples.** (A) PCA plot for taproots; the colors indicate the sample type: field roots, NFR, and FR both analyzed at four storage times (1, 3, 6, and 12 months of root storage). Three biological replicates per sample group are presented. (B) PCA plot for Belgian endive heads; the colors indicate the head samples analyzed at four root storage times (1, 3, 6, and 12 months of root storage). Three biological replicates per sample group are presented.

The forcing process significantly mobilizes the carbohydrates reserves in chicory roots, as shown here and in earlier studies. However, the effect of forcing and storage duration on the profile of secondary metabolites of the Belgian endive is not well studied. Untargeted LCMS-based metabolomics analysis was performed to see the effect of the storage and forcing on the metabolite fingerprints. The PCA of the root samples based on the relative levels of 554 putative compounds indicated clear differences between samples of field roots, NFR, and FR. The PCA showed that the main variation (PC1, 29.5%) in the data set is caused by the process of forcing (Figure 4A). The effect of time of root storage on the metabolite profile in both FR and NFR is visible along the PC1 and PC2. The PCA of the head samples (Figure 4B), based on 295 putative compounds, indicated a clear effect of the root storage duration on the metabolite composition of the head. The main variation (PC1, 33.3%) is caused by differences between heads forced on 12 month stored roots and those that were stored for shorter periods. PC2 explains 19.9% of the variation and indicates differences in metabolite profile between the heads forced on roots that were stored for 1 month and those that were stored for longer periods.

**Figure 5:**
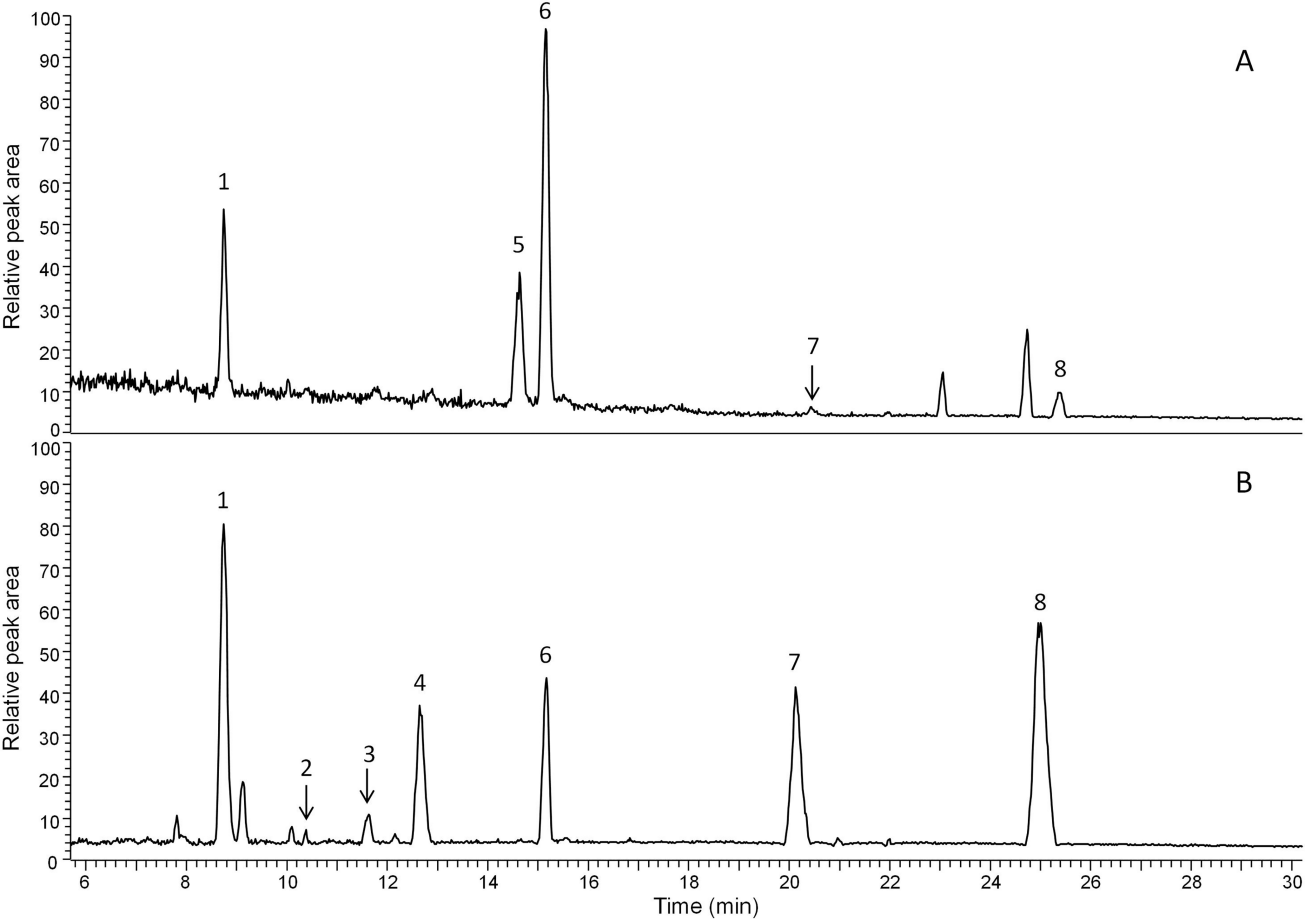
**LCMS profiles of Belgian endive tissues**. (A) LC–MS profile of a Belgian endive head and (B) a NFR are shown. Major STLs and phenolic peaks are labeled: 1) chlorogenic acid, 2) dihydrolactucin, 3) dihydrolactucin 15-oxalate, 4) lactucin 15-oxalate, 5) chicoric acid, 6) isochlorogenic acid A, 7) 8-deoxylactucin 15-oxalate, and 8) lactucopicrin 15-oxalate. *X*-axis: chromatographic retention time (min); Y-axis: MS detector response of the base peak in the negative ionization mode (100% corresponds to 4.00 × 10^7^ ion counts/scan).

Next, targeted quantitative analysis of phenolic compounds and STLs was performed. The major phenolic compounds identified in roots and heads of Belgian endive were chlorogenic acid, isochlorogenic acid A, caftaric acid, and chicoric acid, and the major STLs found were lactucin, dihydrolactucin, lactucopicrin, lactucopicrin 15-oxalate, lactucin 15-oxalate, dihydrolactucin 15-oxalate, and 8-deoxylactucin 15-oxalate. For example, LC–MS profiles of the Belgian endive head and NFR are shown in Figure 5.

Next, the amount of chlorogenic acid, isochlorogenic acid A, caftaric acid, and chicoric acid was quantified (Table 1). The levels of caftaric and chicoric acid were found to be higher in the heads than in the root tissues. This finding is in accordance with an earlier study on phenolics in leaves and roots of industrial chicory, in which higher levels of caftaric and chicoric acid were found in the aerial part of the plant as compared to the roots.^13^ In contrast to the observation that industrial chicory leaves (var. *sativum*) contain low levels of chlorogenic acid and isochlorogenic acid A, both taproots and heads of Belgian endive (var. *foliosum*) accumulate significant levels of these compounds in our study. The levels of most phenolic compounds in the taproots were not influenced by forcing, with the exception of isochlorogenic acid A, which showed between a 2.1 and 4.6 fold increase upon forcing at different time points. Forcing may have induced conversion of the activity of the so far uncharacterized enzyme in chicory,^13^ which converts chlorogenic acid into isochlorogenic acid A; however, only a small reduction in chlorogenic acid was observed.

**Table 1:** Phenolic Compound Levels in Belgian Endive Tissues From the Field and During Storage and Forcing^a^

samples	caftaric acid μg/g DW	chlorogenic acid μg/g DW	chicoric acid μg/g DW	isochlorogenic acid A μg/g DW
**field leaves**	378.5 ± 25.6	666.4 ± 15.7	1085.7 ± 12.2	114.4 ± 6.6
**field roots**	2.6 ± 1.1	469.4 ± 78.1	31.9 ± 5.1	97.9 ± 45.9
**NFR,*t*** = **1**	6.0 ± 1.7 a	615.6 ± 34.7 a	31.9 ± 7.2 a	125.1 ± 30.7 a
**NFR,*t*** = **3**	4.4 ± 0.5 ab	554.9 ± 62.6 a	23.1 ± 3.5 a	50.3 ± 22.4 b
**NFR,*t*** = **6**	2.6 ± 0.6 b	590.0 ± 68.5 a	19.7 ± 3.6 a	128.6 ± 11.8 a
**NFR,*t*** = **12**	3.6 ± 0.7 ab	581.2 ± 34.7 a	34.2 ± 6.4 a	107.8 ± 10.1 a
**FR,*t*** = **1**	3.4 ± 0.6 d	503.6 ± 50.4 d	46.0 ± 4.4 d	307.4 ± 43.8 d
**FR,*t*** = **3**	2.3 ± 0.7 d	500.9 ± 20.4 d	36.8 ± 8.3 d	231.8 ± 6.0 d
**FR,*t*** = **6**	2.7 ± 0.4 d	442.4 ± 3.5 d	34.8 ± 3.4 d	265.4 ± 14.0 d
**FR,*t*** = **12**	3.6 ± 0.9 d	504.2 ± 58.4 d	47.7 ± 10.6 d	337.3 ± 2.8 d
**heads,*t*** = **1**	81.4 ± 1.5 g	313.3 ± 26.2 g	426.1 ± 18.6 g	286.1 ± 33.7 g
**heads,*t*** = **3**	73.3 ± 7.5 g	451.6 ± 20.1 h	357.7 ± 40.8 g	328.1 ± 7.9 g
**heads,*t*** = **6**	74.7 ± 12.0 g	304.1 ± 22.7 g	251.6 ± 52.8 g	269.0 ± 12.8 g
**heads,*t*** = **12**	134.4 ± 14.8 g	305.6 ± 69.2 g	420.5 ± 93.0 g	266.4 ± 60.2 g

a Means and errors are given from triplicate samples. As shown in Supporting Table S3, two-way ANOVA was performed to reveal the interaction between the tissue type and storage time for these compounds. The letters in the table indicate the significance of the Bonferroni posthoc tests, focusing on differences between samples of the same tissue type only.

**Figure 6:**
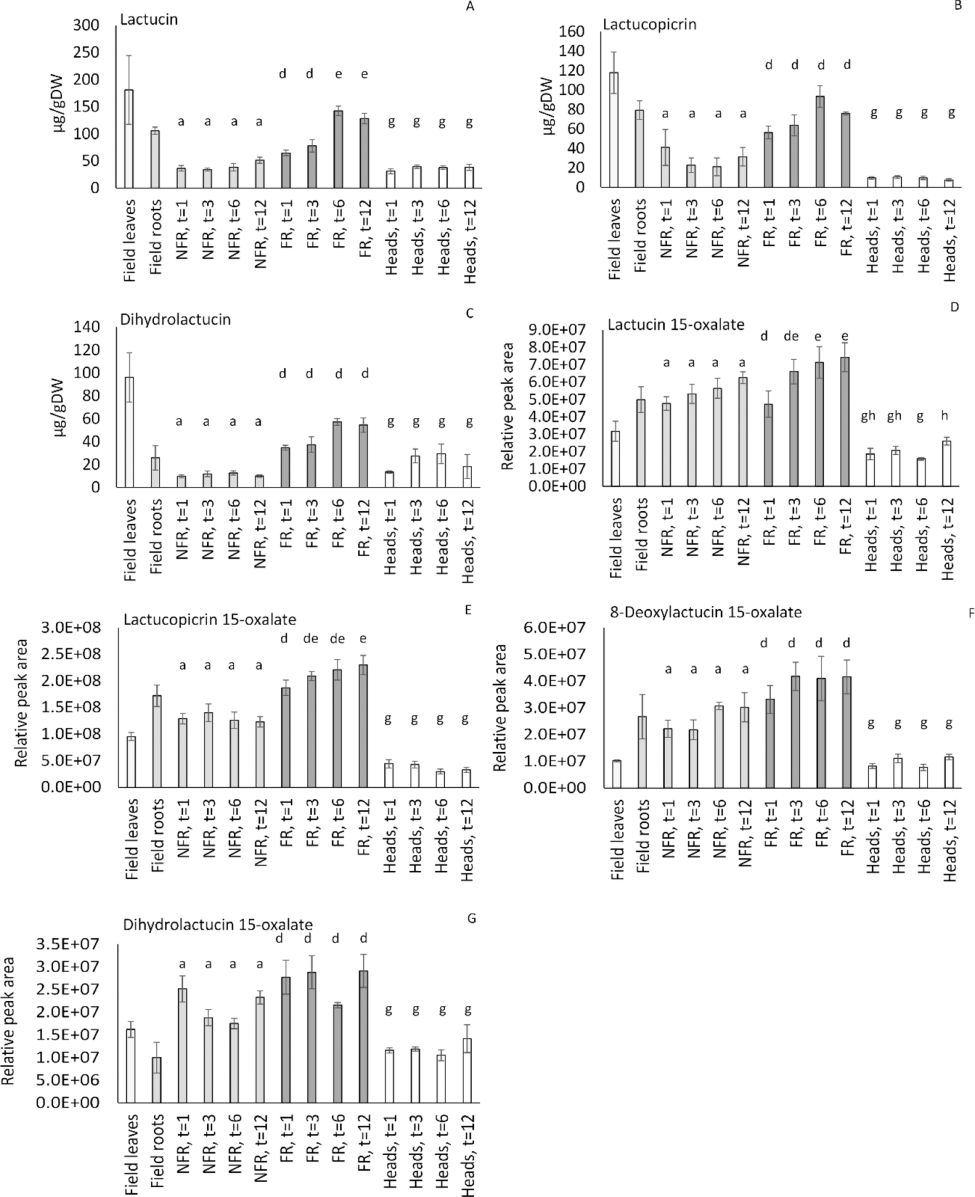
**Sesquiterpene lactone levels in Belgian endive tissues from the field and during storage and forcing.** Sesquiterpene lactone levels in field leaves and roots, NFR, FR, and heads analyzed at four storage times (1, 3, 6, and 12 months of taproot storage). (A) lactucin (μg/g DW), (B) lactucopicrin (μg/g DW), (C) dihydrolactucin (μg/g DW), (D) lactucin 15-oxalate (relative peak area), (E) lactucopicrin 15-oxalate (relative peak area), (F) 8-deoxylactucin 15-oxalate (relative peak area) and (G) dihydrolactucin 15-oxalate (relative peak area). Means and errors are given from triplicate samples. The letters in the figure indicate the significance of the Bonferroni posthoc tests focusing on differences between samples of the same tissue type only.

Quantification was also performed for seven major STLs (Figure 6). The amount of lactucin, dihydrolactucin, lactucopicrin, and lactucopicrin 15-oxalate was found to be significantly increased in the taproots upon forcing on roots stored for 6 months and longer, while lactucin 15-oxalate, dihydrolactucin 15-oxalate, and 8-deoxylactucin 15-oxalate were not significantly altered. In the Belgian endive heads, the levels of all seven STLs were significantly lower compared to the FR. In general, low levels of STLs in the head are often considered as beneficial for consumer appreciation since STLs such as lactucin and lactucopicrin are bitter-tasting compounds.^50,51^

The effect of the root storage duration on the phenolics and sesquiterpene lactone content of taproots was studied in more detail in NFR, FR, and the resulting head. In the NFR, the amount of chlorogenic acid, isochlorogenic acid A, caftaric acid, and chicoric acid was not significantly changed from the time of field harvest up to the 12 months of storage (Table 1 and Supporting Table S4). The level of the STLs in taproots was affected by cold storage (Figure 6). The levels of lactucin, dihydrolactucin, and lactucopicrin were significantly lower in stored roots as compared to the original field roots. This decrease was already observed within the first month after storage. In contrast, the oxalated forms of the STLs were increased or did not significantly differ from the levels detected in the field roots (Figure 6, Supporting Information table S4). At different timepoints during storage, root samples were transferred to the hydroponic culture and forced to produce the heads. The levels of phenolic compounds in the FR were not affected upon prolonged storage of the roots (Table 1). The levels of lactucin, lactucin 15-oxalate, and lactucopicrin 15-oxalate were found to be increased in FR upon prolonged storage of the taproots and significantly different between 1 month and 12 months of storage. It was shown that specific STLs are stronger antifeedants than others and may be specifically produced under feeding stress.^26^ Possibly, other stresses induce certain classes of STLs as well, as we show here for cold storage. Next, the levels of the secondary metabolites were studied in the Belgian endive head in relation to prolonged storage of their taproots. In contrast to the PCA analysis, the targeted analysis shows that levels of major phenolic compounds in the heads were not largely altered upon prolonged root storage (Table 1). The levels of major STLs in the head similarly remained constant upon prolonged root storage.

In order to investigate whether the findings from our analysis on the Belgian endive cultivar “Sweet Lady” would also hold true for another Belgian endive cultivar, the cultivar ‘Flexine’ was taken along in this study. Taproots of the cultivar “Flexine” were stored and forced after 12 months of storage rather than at different time points, as described for “Sweet Lady.” The targeted metabolomics analysis of ‘Flexine’ revealed a comparable overall effect of forcing on the target metabolites and a significant increase in the taproot upon forcing of isochlorogenic acid A, lactucin, and lactucopicrin 15-oxalate, as was found for “Sweet Lady” (Table 2 and Supporting Table S5).

**Table 2:** Phenolic and Sesquiterpene Levels in NFR, FR, and Heads in the Cultivars “Sweet Lady” and “Flexine”^a^

samples	NFR, “Sweet Lady”	NFR, “Flexine”	FR, “Sweet Lady”	FR, “Flexine”	heads, “Sweet Lady”	heads, “Flexine”
caftaric acid (μg/g DW)	3.6 ± 0.7 a	1.7 ± 0.2 b	3.6 ± 0.9 d	1.5 ± 0.1 e	134.4 ± 14.8 g	57.6 ± 3.5 h
chlorogenic acid (μg/g DW)	581.1 ± 34.7 a	573.4 ± 38.5 a	504.2 ± 58.4 d	416.7 ± 19.9 d	305.6 ± 69.2 g	205.6 ± 10.2 g
chicoric acid (μg/g DW)	31.2 ± 34.2 a	13.6 ± 17.5 a	47.7 ± 10.6 d	29.5 ± 3.3 d	420.5 ± 93.0 g	215.0 ± 18.5 h
isochlorogenic acid A (μg/g DW)	107.8 ± 10.1 a	125.2 ± 14.6 a	337.3 ± 2.8 d	337.4 ± 2.2 e	266.4 ± 60.2 g	221.8 ± 45.0 g
lactucin (μg/g DW)	51.3 ± 5.5 a	74.1 ± 11.9 a	128.3 ± 9.6 d	265.7 ± 22.5 e	38.2 ± 5.8 g	82.3 ± 17.9 h
dihydrolactucin (μg/g DW)	10.0 ± 1.0 a	10.0 ± 0.5 a	54.5 ± 6.2 d	57.1 ± 4.4 d	18.2 ± 10.4 g	39.0 ± 2.7 h
lactucopicrin (μg/g DW)	31.5 ± 9.5 a	32.2 ± 11.0 b	75.8 ± 1.5 d	86.7 ± 6.3 e	7.7 ± 1.2 g	19.5 ± 12.0 g
lactucin 15-oxalate (relative peak area)	6.25 × 10^7^ ± 3.36 E + 06 a	1.06 × 10^8^ ± 3.09 E + 06 b	7.43 × 10^7^ ± 8.33 E + 06 d	1.65 × 10^8^ ± 1.52 E + 07 e	2.60 × 10^7^ ± 2.28 E + 06 g	3.99 × 10^7^ ± 9.14 E + 06 g
dihydrolactucin 15-oxalate (relative peak area)	2.33 × 10^7^ ± 1.46 E + 06 a	2.85 × 10^7^ ± 1.30 E + 06 a	2.91 × 10^7^ ± 3.63 E + 06 d	3.77 × 10^7^ ± 2.54 E + 06 d	1.42 × 10^7^ ± 3.07 E + 06 g	1.78 × 10^7^ ± 4.59 E + 06 g
8-deoxylactucin 15-oxalate (relative peak area)	3.02 × 10^7^ ± 5.44 E + 06 a	5.21 × 10^7^ ± 2.77 E + 06 a	4.16 × 10^7^ ± 6.33 E + 06 d	7.02 × 10^7^ ± 9.45 E + 06 d	1.16 × 10^7^ ± 1.06 E + 06 g	1.59 × 10^7^ ± 5.03 E + 06 g
lactucopicrin 15-oxalate (relative peak area)	1.23 × 10^8^ ± 9.79 E+06 a	1.61 × 10^8^ ± 4.67 E+06 a	2.30 × 10^8^ ± 1.79 E+07 d	2.62 × 10^8^ ± 1.52 E+07 d	3.28 × 10^7^ ± 4.75 E + 06 g	4.50 × 10^7^ ± 1.75 E + 07 g

a Values in μg/g DW or relative peak area. Means and errors are given from triplicate samples. As shown in Supporting Table S5, two-way ANOVA was performed to reveal the interaction between the tissue type and storage time for these compounds. The letters in the figure indicate the significance of the Bonferroni posthoc tests, focusing on differences between samples of the same tissue type only.

A comparison of the heads of the two varieties revealed that the cultivar ‘Flexine’ accumulated two times less caftaric acid and about two times less chicoric acid, while the levels of chlorogenic acid and isochlorogenic acid A were not significantly different. STLs levels were comparable for the two cultivars, and only lactucin and dihydrolactucin were accumulated two times more in the cultivar “Flexine.” Previous work on the comparison of Belgian endive cultivars, different from the ones studied here, showed a maximum 3-fold difference in lactucin-like STLs and a maximum 4-fold difference in lactucopicrin-like STLs in the heads of 13 cultivars.^4^

In order to study the effect of the location of cultivation and forcing on the metabolite composition, a second location was included in this study. The cultivar “Sweet Lady” was grown, stored, and successively forced in Beitem, Belgium, in a comparable setup as in location Herent. At this location, the taproots were stored for 6 months and then forced. The stored roots, FR, and heads were collected and included in this analysis. The conclusion from the targeted analysis of the phenolic compounds and STLs was that for these three tissues, most of the studied compounds were not significantly different between the two locations, with the exception of a significantly lower level (50%) of dihydrolactucin in heads harvested al the location Beitem (Table 3 and Supporting Table S6 for statistics). In an earlier study, it was shown that the cultivation location can have a pronounced effect.^4^ This latter study was performed on many more cultivars and five different locations, giving the opportunity to better study the effect in detail. It could also be that the two crop cultivation facilities used in the present study are more comparable to each other than the locations used in the study by Peters et al.

**Table 3:** Phenolic and Sesquiterpene Levels in NFR, FR, and Heads Analyzed in Two Growing Locations^a^

samples	NFR, Herent	NFR, Beitem	FR, Herent	FR, Beitem	heads, Herent	heads, Beitem
caftaric acid (μg/g DW)	2.6 ± 0.6 qa	2.2 ± 0.1 a	2.7 ± 0.4 d	3.5 ± 0.8 d	74.7 ± 12.0 g	72.6 ± 15.3 g
chlorogenic acid (μg/g DW)	590.0 ± 68.5 a	601.4 ± 83.9 a	442.4 ± 3.5 d	418.4 ± 60.2 d	304.1 ± 22.7 g	246.6 ± 27.7 g
Chicoric acid (μg/g DW)	14.7 ± 19.7 a	17.8 ± 24.8 a	34.8 ± 3.4 d	39.9 ± 7.5 d	251.6 ± 52.8 g	324.5 ± 52.4 g
isochlorogenic acid A(μg/g DW)	128.6 ± 11.8 a	114.5 ± 29.4 a	265.4 ± 14.0 d	308.5 ± 40.7 d	269.0 ± 12.8 g	204.9 ± 14.2 g
lactucin (μg/g DW)	38.4 ± 7.0 a	41.3 ± 6.3 a	142.5 ± 8.7 d	192.9 ± 16.5 qd	37.6 ± 3.3 g	31.9 ± 4.3 g
dihydrolactucin (μg/g DW)	12.6 ± 1.9 a	15.9 ± 1.3 a	57.2 ± 3.1 d	63.0 ± 10.0 d	29.4 ± 8.6 g	15.1 ± 1.0 h
lactucopicrin (μg/g DW)	21.2 ± 9.1 a	30.0 ± 10.6 a	93.4 ± 11.1 d	116.3 ± 15 d	9.8 ± 1.7 g	6.6 ± 1.3 g
lactucin 15-oxalate (relative peak area)	5.64 × 10^7^ ± 5.68 E + 06 a	5.47 × 10^7^ ± 7.48 E+06 a	7.13 × 10^7^ ± 9.12 E+06 d	8.43 × 10^7^ ± 1.46 E+07 d	1.59 × 10^7^ ± 8.04 E+05 g	1.69 × 10^7^ ± 2.30 E+06 g
dihydrolactucin 15-oxalate (relative peak area)	1.75 × 10^7^ ± 1.12 E + 06 a	1.84 × 10^7^ ± 1.57 E + 06 a	2.16 × 10^7^ ± 5.87 E + 05 d	1.64 × 10^7^ ± 3.07 E + 06 d	1.05 × 10^7^ ± 1.21 E + 06 g	9.93 × 10^6^ ± 8.60 E + 05 g
8-deoxylactucin 15-oxalate (relative peak area)	3.07 × 10^7^ ± 1.46 E + 06 a	3.14 × 10^7^ ± 6.69 E + 06 a	4.10 × 10^7^ ± 8.33 E + 06 d	5.72 × 10^7^ ± 1.29 E + 07 d	7.71 × 10^6^ ± 1.19 E + 06 g	6.70 × 10^6^ ± 8.12 E + 05 g
lactucopicrin 15-oxalate (relative peak area)	1.26 × 10^8^ ± 1.51 E + 07 a	1.45 × 10^8^ ± 1.08 E + 07 a	2.21 × 10^8^ ± 1.93 E + 07 d	2.95 × 10^8^ ± 3.80 E + 07 d	2.93 × 10^7^ ± 4.77 E + 06 g	2.40 × 10^7^ ± 3.52 E + 06 g

a Values in μg/g DW or relative abundance. Means and errors are given from triplicate samples. As shown in Supporting Table S6, two-way ANOVA was performed to reveal the interaction between the tissue type and storage time for these compounds. The letters in the figure indicate the significance of the Bonferroni posthoc tests, focusing on differences between samples of the same tissue type only. Pairwise comparisons to field leaves and roots are discussed in the main text but not presented here.

This study demonstrates a stable carbohydrate composition of Belgian endive heads throughout the cultivation season. Additionally, the composition of major STLs and phenolics, important for the bitter taste of the heads, remains largely unaltered during the entire cultivation season until up to 12 months. Importantly, this study also demonstrates that the Belgian endive roots are potentially a valuable source of carbohydrates, terpenes, and phenolics. This highlights the potential of Belgian endive roots for valorization, whereas they are currently largely unused, and 400.000 tons are discarded in the EU each year. Further studies into the extraction and processing of the roots, as well as on bioactivity of extracted compounds, are needed to support the development of valorization strategies.

## Supporting Information Available

Statistical significance test for carbohydrate analysis; statistical analysis of carbohydrate analysis performed for the comparison of the field roots and the stored roots; statistical significance test for secondary metabolite analysis; statistical analysis of secondary metabolite analysis performed for the comparison of the field roots and the stored roots; statistical significance test for secondary metabolite analysis of the cultivar comparison; and statistical significance test for secondary metabolite analysis of the location comparison (PDF)
