# Supplementary material for: Effect of Root Storage and Forcing on the Carbohydrate and Secondary Metabolite Composition of Belgian Endive (Cichorium intybus L. Var. foliosum)
Source: ACS Food Sci Technol. 2022 Sep 23;2(10):1546–57. doi: 10.1021/acsfoodscitech.2c00182 (PMC9594316; doi:10.1021/acsfoodscitech.2c00182)
Supplement: Supplementary file 1 — fs2c00182_si_001.pdf [file fs2c00182_si_001.pdf]

Supplementary data to:

Effect of Root Storage and Forcing on the Carbohydrate and Secondary Metabolite Composition of Belgian Endive (*Cichorium intybus* L. var. *foliosum*)

Jeroen van Arkel[a], Anna Twarogowska[b], Yannah Cornelis[c], Tania De Marez[d], Jasper Engel[a], Peter Maenhout[d], Ric C.H. de Vos[a], Jules Beekwilder[a], Bart Van Droogenbroeck[b] and Katarina Cankar[a]*

[a] Wageningen University and Research, Wageningen Plant Research, BU Bioscience, Droevendaalsesteeg 1, 6708PB Wageningen, The Netherlands

[b] ILVO, Flanders Research Institute for Agriculture, Fisheries and Food, Technology and Food Science Unit, Brusselsesteenweg 370, BE-9090 Melle, Belgium

[c] Praktijkpunt Landbouw Vlaams-Brabant vzw, Blauwe Stap 25, BE-3020 Herent, Belgium

[d] Inagro vzw, Ieperseweg 87, BE-8800, Rumbeke-Beitem, Belgium

***corresponding author: katarina.cankar@wur.nl**

Supplementary table S1: **Statistical significance test for carbohydrate analysis**

The level of statistical significance of the effects of storage time, tissue and their interaction in selected compounds as assessed by logistic regression in combination with a Wald test. Significant effects ($p < 0.05$) are indicated in bold. The mDP that was assessed by two-way ANOVA (type 3 F-test). Significant effects ($p < 0.05$) are indicated in bold.

**Dependent variable**	**Time**	**Tissue**	**Time*Tissue**
Total carbohydrate	0.195	**< 0.001**	0.263
Free fructose	0.476	**< 0.001**	**< 0.001**
Free sucrose	**0.001**	**< 0.001**	**0.023**
mDP	**< 0.001**	**< 0.001**	**< 0.001**
Free glucose	0.072	**< 0.001**	**0.001**
Inulin	0.044	**< 0.001**	**0.003**

Supplementary table S2: Statistical analysis of carbohydrate analysis performed for the comparison of the field roots and the stored roots

Statistical comparison of the field roots as control group and the stored roots as sample groups using respectively ANOVA and Wald test for respectively the mDP and the other variables, in combination with Dunnett's Post Hoc test. Significant effects ($p < 0.05$) are indicated in bold**.**

Multiple Comparisons to control group: Field roots					
**Dunnett t (2-sided) a**					
**Dependent variable**	**Comparison**	**Mean Difference**	**95% Confidence Interval**		**P. (significance)**
			**Lower Bound**	**Upper Bound**	
Total Carbohydrate	NFR, t=1	-0.018	-0.152	0.116	0.959
	NFR, t=3	-0.037	-0.171	0.096	0.792
	NFR, t=6	-0.042	-0.176	0.092	0.735
	NFR, t=12	-0.126	-0.259	0.007	0.063
Free Fructose	NFR, t=1	-0.306	-0.531	-0.081	**0.010**
	NFR, t=3	-0.208	-0.427	0.011	0.064
	NFR, t=6	-0.391	-0.621	-0.161	**0.002**
	NFR, t=12	0.319	0.124	0.515	**0.003**
Free Sucrose	NFR, t=1	0.873	0.463	1.283	**< 0.001**
	NFR, t=3	1.199	0.803	1.596	**< 0.001**
	NFR, t=6	1.073	0.672	1.474	**< 0.001**
	NFR, t=12	1.375	0.985	1.766	**< 0.001**
mDP	NFR, t=1	-2.513	-2.748	-2.278	**< 0.001**
	NFR, t=3	-3.683	-3.918	-3.448	**< 0.001**
	NFR, t=6	-3.553	-3.788	-3.318	**< 0.001**
	NFR, t=12	-4.137	-4.372	-3.902	**< 0.001**
Free Glucose	NFR, t=1	0.310	-0.163	0.784	0.235
	NFR, t=3	0.549	0.096	1.001	**0.019**
	NFR, t=6	0.512	0.056	0.968	0.028
	NFR, t=12	0.800	0.366	1.235	**0.001**
Inulin	NFR, t=1	-0.274	-0.449	-0.098	**0.004**
	NFR, t=3	-0.499	-0.676	-0.322	**< 0.001**
	NFR, t=6	-0.398	-0.574	-0.222	**< 0.001**
	NFR, t=12	-0.810	-0.990	-0.629	**< 0.001**

Supplementary table S3: Statistical significance test for secondary metabolite analysis

The level of statistical significance of the effects of storage time, tissue and their interaction in selected compounds, as assessed by two-way ANOVA (type 3 F-test). Significant effects ($p < 0.05$) are indicated in bold.

**Dependent variable**	**Time**	**Tissue**	**Time*Tissue**
Caftaric acid	**0.006**	**< 0.001**	**0.019**
Chlorogenic acid	0.096	**< 0.001**	**0.029**
Chicoric acid	**< 0.001**	**< 0.001**	0.835
Isochlorogenic acid A	0.011	**< 0.001**	**0.003**
Lactucin	**< 0.001**	**< 0.001**	**0.001**
Lactucopicrin	0.916	**< 0.001**	0.078
Dihydrolactucin	**0.006**	**< 0.001**	0.127
Lactucin 15-oxalate	**< 0.001**	**< 0.001**	**0.039**
Lactucopicrin 15-oxalate	0.233	**< 0.001**	**0.036**
8-deoxylactucin 15-oxalate	**0.018**	**< 0.001**	0.065
Dihydrolactucin 15-oxalate	**0.001**	**< 0.001**	0.334

Supplementary table S4: **Statistical analysis of secondary metabolite analysis performed for the comparison of the field roots and the stored roots**

Statistical analysis on STLs and Phenolics for the comparison of the field roots (control group) and the stored roots (sample groups) using ANOVA with Dunnett's Post Hoc. Significant effects ($p < 0.05$) are indicated in bold**.**

Multiple Comparisons to control group: Field roots					
Dunnett t (2-sided) a					
**Dependent variable**	**Comparison**	**Mean Difference**	**95% Confidence Interval**		**P. (significance)**
			**Lower Bound**	**Upper Bound**	
Caftaric acid	NFR, t=1	1.212	0.086	2.339	**0.035**
	NFR, t=3	0.839	-0.288	1.966	0.164
	NFR, t=6	0.035	-1.092	1.162	0.999
	NFR, t=12	0.510	-0.616	1.637	0.515
Chlorogenic acid	NFR, t=1	0.410	-0.063	0.882	0.094
	NFR, t=3	0.253	-0.220	0.725	0.389
	NFR, t=6	0.341	-0.132	0.813	0.182
	NFR, t=12	0.326	-0.146	0.799	0.208
Dihydrolactucin	NFR, t=1	-1.298	-2.194	-0.403	**0.006**
	NFR, t=3	-1.032	-1.927	-0.137	**0.024**
	NFR, t=6	-0.932	-1.827	-0.037	**0.041**
	NFR, t=12	-1.269	-2.164	-0.374	**0.007**
Lactucin	NFR, t=1	-1.548	-2.046	-1.049	**< 0.001**
	NFR, t=3	-1.631	-2.129	-1.133	**< 0.001**
	NFR, t=6	-1.478	-1.976	-0.980	**< 0.001**
	NFR, t=12	-1.047	-1.545	-0.549	**< 0.001**
Dihydrolactucin 15-oxalate	NFR, t=1	1.409	0.672	2.145	**0.001**
	NFR, t=3	0.992	0.255	1.728	**0.010**
	NFR, t=6	0.893	0.157	1.630	**0.018**
	NFR, t=12	1.304	0.568	2.040	**0.001**
Lactucin 15-oxalate	NFR, t=1	-0.053	-0.487	0.380	0.968

	NFR, t=3	0.100	-0.334	0.533	0.869
	NFR, t=6	0.185	-0.249	0.619	0.558
	NFR, t=12	0.340	-0.094	0.774	0.137
Chicoric acid	NFR, t=1	-0.023	-0.832	0.786	0.999
	NFR, t=3	-0.466	-1.275	0.344	0.334
	NFR, t=6	-0.704	-1.513	0.105	0.093
	NFR, t=12	0.095	-0.714	0.904	0.971
Isochlorogenic acid A	NFR, t=1	0.448	-0.976	1.873	0.745
	NFR, t=3	-0.999	-2.424	0.426	0.199
	NFR, t=6	0.533	-0.891	1.958	0.646
	NFR, t=12	0.278	-1.147	1.703	0.908
Lactucopicrin	NFR, t=1	-0.202	-1.057	0.653	0.860
	NFR, t=3	-0.233	-1.088	0.623	0.810
	NFR, t=6	0.278	-0.577	1.134	0.728
	NFR, t=12	0.234	-0.621	1.090	0.808
Lactucopicrin 15-oxalate	NFR, t=1	-1.117	-2.644	0.409	0.174
	NFR, t=3	-1.859	-3.386	-0.333	**0.018**
	NFR, t=6	-2.015	-3.542	-0.488	**0.011**
	NFR, t=12	-1.400	-2.927	0.127	0.074
8-deoxylactucin 15-oxalate	NFR, t=1	-0.202	-1.057	0.653	0.860
	NFR, t=3	-0.233	-1.088	0.623	0.810
	NFR, t=6	0.278	-0.577	1.134	0.728
	NFR, t=12	0.234	-0.621	1.090	0.808

Supplementary table S5: **Statistical significance test for secondary metabolite analysis of the cultivar comparison**

The level of statistical significance of the effects of the cultivar, tissue and their interaction in selected compounds, as assessed by two-way ANOVA (type 3 F-test). Significant effects ($p < 0.05$) are indicated in bold.

**Dependent variable**	**Cultivar**	**Tissue**	**Cultivar * Tissue**
Caftaric acid	**< 0.001**	**< 0.001**	0.897
Chlorogenic acid	**0.019**	**< 0.001**	0.157
Chicoric acid	**< 0.001**	**< 0.001**	**0.678**
Isochlorogenic acid A	0.914	**< 0.001**	0.308
Lactucin	**< 0.001**	**< 0.001**	0.122
Lactucopicrin	0.140	**< 0.001**	0.259
Dihydrolactucin	**0.032**	**< 0.001**	**0.027**
Lactucin 15-oxalate	**< 0.001**	**< 0.001**	0.087
Lactucopicrin 15-oxalate	**0.045**	**< 0.001**	0.814
8-deoxylactucin 15-oxalate	**0.001**	**< 0.001**	0.447
Dihydrolactucin 15-oxalate	**0.022**	**< 0.001**	0.956

Supplementary table S6: Statistical significance test for secondary metabolite analysis of the location comparison

The level of statistical significance of the effects of location, tissue and their interaction in selected compounds, as assessed by two-way ANOVA (type 3 F-test). Significant effects ($p < 0.05$) are indicated in bold.

**Dependent variable**	**Location**	**Tissue**	**Location * Tissue**
Caftaric acid	0.819	**< 0.001**	0.416
Chlorogenic acid	0.191	**< 0.001**	0.356
Chicoric acid	0.077	**< 0.001**	0.866
Isochlorogenic acid A	0.244	**< 0.001**	0.106
Lactucin	0.366	**< 0.001**	0.062
Lactucopicrin	0.742	**< 0.001**	0.145
Dihydrolactucin	0.339	**< 0.001**	**0.009**
Lactucin 15-oxalate	0.439	**< 0.001**	0.575
Lactucopicrin 15-oxalate	0.146	**< 0.001**	0.121
8-deoxylactucin 15-oxalate	0.542	**< 0.001**	0.209
Dihydrolactucin 15-oxalate	0.321	**< 0.001**	0.057
